# Global network centrality of university rankings

**DOI:** 10.1098/rsos.171172

**Published:** 2017-10-04

**Authors:** Weisi Guo, Marco Del Vecchio, Ganna Pogrebna

**Affiliations:** 1 School of Engineering, University of Warwick, Warwick, UK; 2 Department of Statistics, University of Warwick, Warwick, UK; 3 Warwick Manufacturing Group, University of Warwick, Warwick, UK; 4 The Alan Turing Institute, London, UK; 5 Birmingham Business School, University of Birmingham, Birmingham, UK

**Keywords:** university performance, complex network, transport network

## Abstract

Universities and higher education institutions form an integral part of the national infrastructure and prestige. As academic research benefits increasingly from international exchange and cooperation, many universities have increased investment in improving and enabling their global connectivity. Yet, the relationship of university performance and its global physical connectedness has not been explored in detail. We conduct, to our knowledge, the first large-scale data-driven analysis into whether there is a correlation between university relative ranking performance and its global connectivity via the air transport network. The results show that local access to global hubs (as measured by air transport network *betweenness*) strongly and positively correlates with the ranking growth (statistical significance in different models ranges between 5% and 1% level). We also found that the local airport’s aggregate flight paths (*degree*) and capacity (*weighted degree*) has no effect on university ranking, further showing that global connectivity distance is more important than the capacity of flight connections. We also examined the effect of local city economic development as a confounding variable and no effect was observed suggesting that access to global transportation hubs outweighs economic performance as a determinant of university ranking. The impact of this research is that we have determined the importance of the centrality of global connectivity and, hence, established initial evidence for further exploring potential connections between university ranking and regional investment policies on improving global connectivity.

## 1. Introduction

Universities have existed for over 1000 years, and represent a community of academics, teachers, students and administrators.^1^ Today, over 26 000 higher education institutions exist worldwide. Higher education remains one of the strongest growing and most resilient economic sectors. In the UK alone, universities generate over *$*40 billion (3% of GDP) and employ 3% of the workforce. As a result, universities form a part of key national and regional infrastructure.

Over the past decade, there is growing international competition for and cooperation between the brightest minds. Globally connected universities have acted as the primary vehicle for this process [1]. At the sector level, this has led to increased competition and cooperation in science, technology and innovation (STI). Some drivers include the emergence of BRICS (Brazil, Russia, India, China and South Africa) countries as new scientific powers, global human challenges such as climate change, and the general globalization of research and development funding [2]. Individually, it has been shown that international cooperation leads to higher research impact and citations [3], and self-organized preferential attachment behaviour allows greater flexibility in collaborations [4]. While some universities have risen steadily in both research and teaching, others have experienced a continued decline in performance. Understanding the underlying factors that drive university performance is important. Over the past decade, universities have recognized the need to improve both digital connectivity [5] and physical *connectedness* to the world [1]. Universities certainly recognize the importance of research and teaching collaboration as well as effective leverage on emerging economies. For example, many universities have established overseas alliances, partnerships and joint campuses. Aside from exploiting the benefits of new economic opportunities [6], there is also the importance of facilitating academic exchange and sharing facilities [7].

In 2007, the President of New York University (NYU), John Sexton, famously argued that universities in globally connected cities will have the potential to eventually supersede rural counterparts. In ‘FIRE and ICE: the Knowledge Century and the Urban University’, the premise is that global cities are conventionally focused on the modern forms of commerce: finance, insurance, and real estate (FIRE). There are comprehensive case studies which detail how many of the global FIRE cities today did not previously have an equally strong higher education sector (Singapore, Hong Kong, Sydney, New York, Seoul, etc.) [8]. Their strength in FIRE stems from their strategic position in the global trade. Yet, it is clear that regardless of the FIRE sector’s future, its intellectual, cultural and educational (ICE) sectors are also part of its complete identity to attract talent and complement the FIRE sector. For example, New York City today is a home to 128 Nobel Laureates and continues to be the leading destination for college graduates. The combination and synergy of FIRE and ICE assets will continue to inspire and drive the global urban academic sector (i.e. New York, Singapore, Hong Kong, Shanghai), propelling it to be increasingly competitive against traditional university powerhouses based in less global settlements (i.e. Boston, Cambridge and Oxford). This paper sets out to discover if, in the past decade, the universities in globally connected cities have benefited disproportionately more from their increased international transport connectivity than other universities. While there is literature that shows the importance of international co-authorships in research [9], there are no studies which test for the importance of physical international transport infrastructure and the general excellence of a university (which would take into account research, teaching and service quality). Existing studies on academic research collaborations were conducted without considering physical proximity to transportation hubs or only took into account historical proximity. While this approach is valid for considering research collaborations, many other interactions related to the university performance cannot be assessed in this manner. Therefore, it is important to test the impact of existing physical connectedness of universities and the impact it has on annual performance.

### 1.1. University ranking systems

University rankings, sometimes known as *league tables*, are prepared and published by commercial entities such as media companies and professional societies (in 57.9% of the cases) as well as by governmental agencies (7%) [10]. The three main dimensions taken into account when evaluating an institution are generally teaching, research, and service quality [11]. To conduct such evaluations, a variety of factors have been taken into account by major ranking providers [12]. Since this variety of performance measures has to be condensed into a single scale, ranking systems depend heavily on the methods and weights used. Two of the most common methods are to scale each variable relative to the highest performing entity, and to standardize the variables to remove mean and variance effects [13]. Weight-wise, it has been found that ‘regional weights’ are sometimes mentioned yet never disclosed [14]. These regional weights vary across rankings and tend to reflect the view of the publisher rather than follow an unbiased theoretical approach [15,16].

In order to gauge the performance of universities, we consider an internationally well established higher education ranking which has been ‘cited and employed’ in many academic studies [17]: the Academic Ranking of World Universities (ARWU). The data from ARWU can be used freely for research and its methodology has remained consistent since 2005. We have chosen ARWU ranking not only because it is an open-source ranking but also because it appears to be concerned with overall excellence of universities. Indeed, 80% of the ranking is composed of indicators directly relating to research excellence. However, 20% of factors are related to teaching excellence. Specifically, the ranking takes into account not only the Nobel Prizes and Field Medals of the current staff but also the Nobel Prizes and Field Medals achieved by the Universities’ alumni (see table 1 which presents the components of the ARWU ranking exactly as described at the ARWU website [17]).

**Table 1.** Indicators and their relative weight in the ARWU ranking.

criteria	indicator	code	weight (%)
quality of education	alumni winning Nobel Prizes and Fields Medals	alumni	10
quality of faculty (i)	staff winning Nobel Prizes and Fields Medals	award	20
quality of faculty (ii)	highly cited researchers in 21 subject categories	HiCi	20
research output (i)	papers published in Nature and Science	NS	20
research output (ii)	papers indexed in Science Citation Index-expanded	PUB	20
	and Social Science Citation Index		
*per capita* performance	*per capita* academic performance of an institution	PCP	10
total			100

### 1.2. Global connectivity and economic growth

#### 1.2.1. Air transport network

Global connectivity is often measured by the connectedness of the regional airports (hubs) and accessibility of these hubs. In terms of the air transport network, its complexity has led many researchers to apply network science in order to better understand its properties. In general, airports are represented by nodes and flights are represented by links, weighted by the capacity. A variety of studies at the network level and the node level examined the statistical structure of air transport networks [18,19], and its relation to travel patterns, i.e. average shortest path *d* grows with log⁡(S), where *S* is the number of nodes in the network [18]. In general, the network centrality measures allow us to understand the importance of an airport in not just how it serves as a source or destination of travel, but also allows us to assess its importance as a transfer hub (betweenness) and its hop distance to every other airport in the world (closeness).

#### 1.2.2. Economic growth

Global connectivity of a particular university might be related to the state of economic development of area (city) where this university is located. Therefore, in our analysis, we control for the economic growth at the geographical location of the university. To this end, we employ the Globalization and World Cities (GaWC) Research Network, which ranks over 300 cities based on their transactional economy (i.e. financial investment) in: accountancy, advertising, banking/finance, and law. It ignores culture and education and political factors, and, as such, serves as a good proxy for the economic condition of the city. GaWC allows us to rank cities on a scale from 0 (sufficiency level) to 11 (alpha++ level), where levels have the following meaning according to the 2016 classification^2^ :

— alpha++ (11)—refers to the most developed cities which are most integrated with the global economy (there are only two cities in this category—London and New York City);
— alpha+ (10)—seven highly developed and economically connected cities;
— alpha (9)—19 cities linking highly successful major economic regions into the world economy;
— alpha−(8)—21 cities linking successful major economic regions into the world economy;
— beta+ (7)—24 cities linking highly successful moderate economic regions into the world economy;
— beta (6)—19 cities linking successful moderate economic regions into the world economy;
— beta− (5)—38 cities linking less successful moderate economic regions into the world economy;
— gamma+ (4)—24 cities linking highly successful small economic regions into the world economy;
— gamma (3)—28 cities linking successful small economic regions into the world economy;
— gamma− (2)—32 cities linking less successful small economic regions into the world economy;
— high sufficiency (1)—34 cities having a high degree of accountancy, advertising, banking/finance, and law services and functioning independently of world cities; and
— sufficiency (0)—112 cities which have sufficient development level to not be obviously dependent on world cities.

### 1.3. Hypothesis and objectives

The hypothesis tested in this paper is whether global air transport connectivity improves a university’s relative ranking performance. In order to test this hypothesis, we have collected data from 2005 to 2016 on transport connectivity, university rankings and economic development. Our objectives were to: (i) mine the data on university ranking and gather global air transport data for the time period from 2005 to 2016); (ii) develop appropriate measures for relative university ranking change and network centrality of the transport network within a catchment area; and (iii) statistically test their mutual correlation and examine the effect of cofactors.

## 2. Data and extraction procedure

All our data used in this study are available in [20], which includes (2005–2016): each university’s ranking, location, the nearest city’s economic output and the aggregated neighbouring airports’ network centrality values.

### 2.1. University and global city rankings

University and Global Cities rankings were extracted from the ARWU website. Economic growth data were obtained from the GaWC website. These databases were freely available online for non-commercial use.

**Figure 1.**
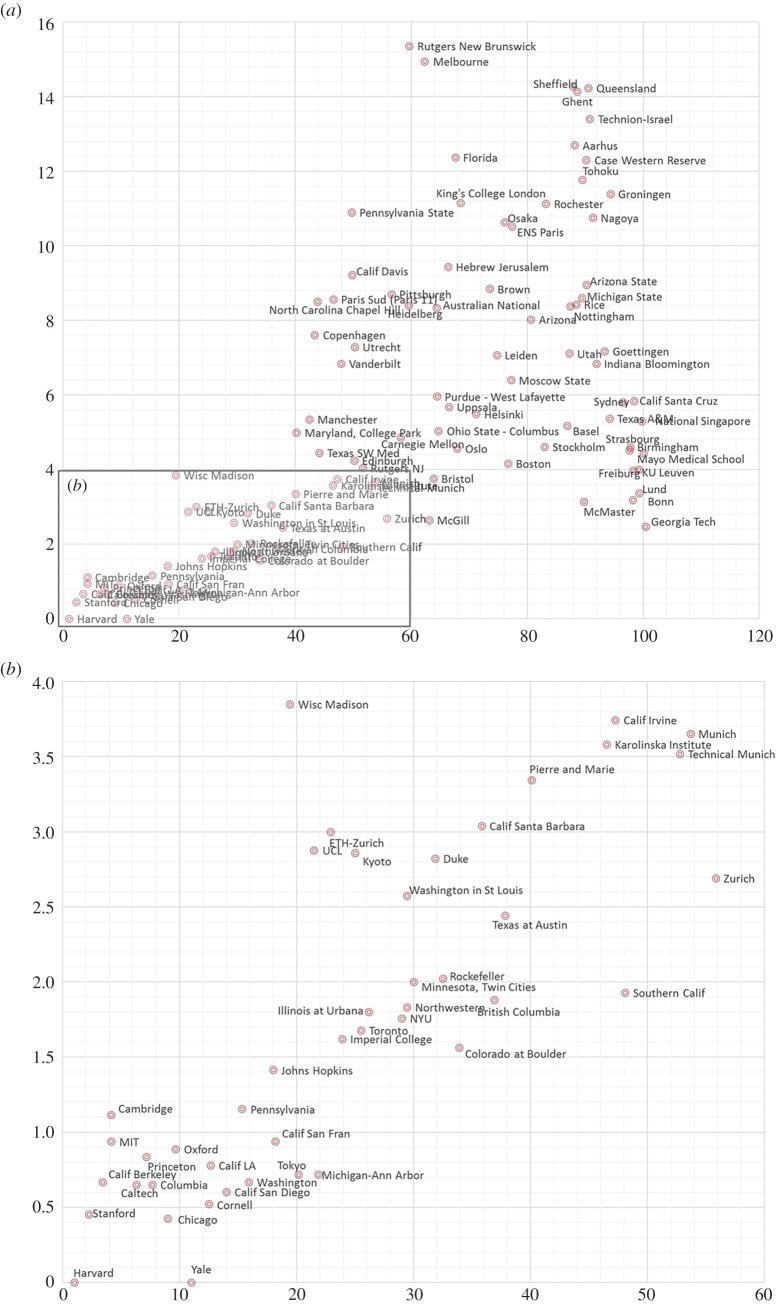
ARWU mean ratings of the top 100 universities plotted versus standard deviation of ranks for each university. (*a*) All top 100 universities, (*b*) zoomed in top 60. Horizontal axis shows the average ranking of each university over 12 years. Vertical axis shows standard deviation of ranks for each university over 12 years.

Web scraping (also called Web harvesting or Web data extraction), an automated technique aimed at obtaining information from the web [21], was used to scrape information from the main ARWU website. For the purpose of this research, we have chosen to exploit web scraping techniques in order to download the ARWU higher education ranking system in the following fashion. Online university rankings come in the form of tables. Let U be the set of root URLs pointing at the different years of the ARWU ranking—where by root we mean the one which points at the beginning of the table. Then, we run the following procedure for each URL in U: 
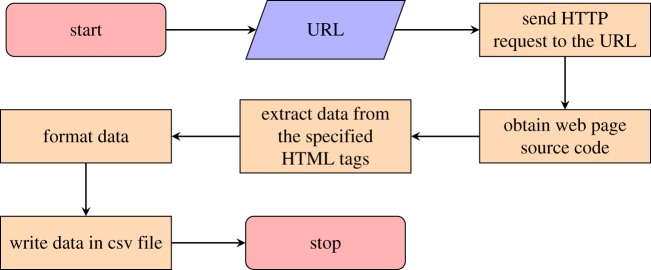


For the sake of rapid prototyping and re-usability, this rationale was implemented in R. The final dataset organized in csv format included data from 642 universities over 12 years (6008 observations). ARWU-ranked universities from 1 (highest rank) to 500 (lowest rank) for each of the years from 2005 to 2016.^3^ The top 100 universities in the ARWU ranking were captured by scalar values between 1 and 100. For the remainder of the rankings, an interval was provided (101–150, 151–200, etc.). Figure 1 shows the average ranking of the top 100 universities which captures their relative quality (horizontal axis) plotted against standard deviation of the ranking which allows us to assess ranking stability for each university (vertical axis).

GaWC ranking was downloaded from the official website and merged with the ARWU database. In the process of data collection, we were aware of two issues: (i) the (scraped) location data associated with each university was not always accurate enough to be correctly merged with the GaWC dataset; and (ii) some universities were located in cities which were not present in the GaWC dataset. We solved the above mentioned problems as follows: (i) we used the Google Maps Geocoding API^4^ which, given the name of a university, returned its full address; (ii) given a university *u*_*i*_ in city *c*_*i*_, where *c*_*i*_ was not in the GaWC dataset, we found the GaWC city closest to the this university and took its ranking as a proxy of the economic growth.

### 2.2. Global air transport

**Figure 2.**
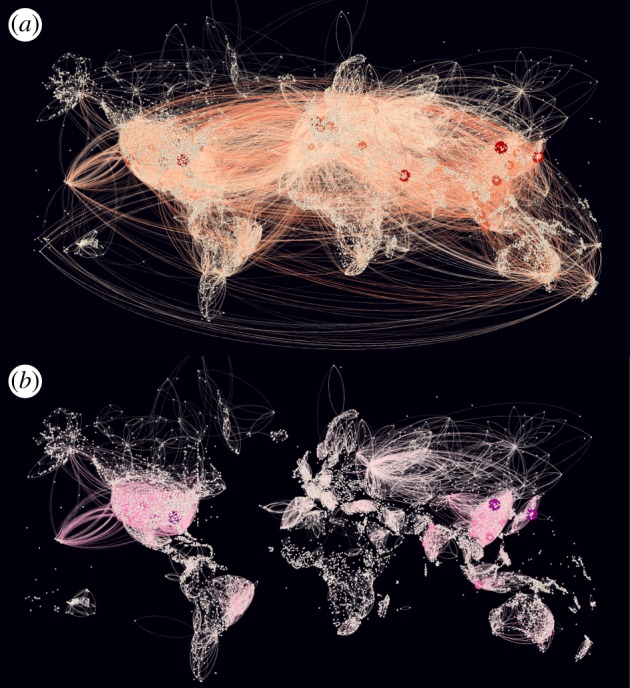
Complex network of city nodes (airports) with directed and weighted air transport links for 2016. Node size reflects weighted degree and link line width indicates number of seats per month. (*a*) Global network comprises 9033 nodes and 101 042 links. (*b*) A number of domestic subgraphs which comprises 9032 nodes and 53 496 links.

The air transport data was purchased from commercial vendor OAG, an air travel intelligence company, and network level data (flights per airport) was calculated.^5^ In our analysis, we used data obtained for the years of 2005–2016. The network-level data (degree, weighted degree, betweenness, closeness, and eigenvector) was merged with the ARWU–GaWC dataset. Approximately speaking (data varies from year to year), there are over 9000 airports globally and over 101 042 unique flight paths connecting these airports. Domestic flights account for approximately 50% of these flight paths. The network which we constructed using this OAG data is shown in figure 2 for (*a*) international and domestic travel, and (*b*) domestic travel only.

## 3. Methods

### 3.1. Relative ranking analysis

In order to be able to correlate the global connectivity of a university with its ranking, we had to capture the variability as well as the trend of its raking across the years. To do so, we used the following approach. Let *M*_*ik*_ be the position of university *i* in year *k* such that 1≤*i*≤*n*,1≤*k*≤*s*. Then, given a university say $\tilde{i}$, we estimated the slope of the trend capturing the change in ranking across the years via the Sen’s estimate [22] and we timed it by the coefficient of variation of $M_{\tilde{i},k}$.

Initially, we computed *S*=*s*(*s*−1)/2 slope estimates:

3.1 $$q=\frac{M_{\tilde{i},k}-M_{\tilde{i},t}}{k-t},$$

for all *k*>*t* where *k*=1,…,*s* and *t*=1,…,*s*−1. Then, the median of these *S* estimates of slope was taken as the non-parametric slope estimate $\beta_{\tilde{i}}$. At this point, we defined the coefficient capturing the overall trend and fluctuation of university $\tilde{i}$ as

3.2 $$\gamma_{\tilde{i}}:=\beta_{\tilde{i}}\frac{\sigma }{\mu },$$

where *σ* and *μ* were the standard deviation and mean of $M_{\tilde{i},k}$, respectively.

### 3.2. Clustered rankings

As we pointed out earlier, the ARWU ranks a total of 500 universities each year, however, while a fine grained ranking position is available for the first 100 entries, the position of the remaining 400 is clustered in groups of 50 (e.g. any university ranked between 101 and 150 will have ranking position 101–150). This is owing to the fact that after the 100th position, the score used to create the ranking does not change enough to justify a fine grained ranking.

As a consequence, we analysed the first 100 entries as well as the entire ranking by considering synthetic rankings generated from sorting the clustered institutions by their scores on the six objective indicators that made up the ranking (table 1).^6^ Additionally, in order to capture effects beyond the top 100 universities, we employed the interval regression analysis which allowed us to use an interval variable as a dependent variable.

### 3.3. Global connectivity analysis

In our analysis, we leveraged on network science [23] to determine the network importance of specific airport hubs, similar to the analysis conducted in [24]. The global connectivity of an airport hub a∈A was determined by its complex network properties. Several network centrality properties will be examined, including: adjustable weighted degree *C*_*a*,*w*_ (number of distinctive connections—including capacity of the links), adjustable weighted betweenness *C*_*a*,*b*_ (number of global shortest flight hop paths—including capacity of the airport), eigenvector centrality *C*_*a*,*e*_ (influence of airports it is connected to) and closeness centrality *C*_*a*,*c*_ (distance to all other cities in the world). The centrality measures *C*_*a*_ are unique to each airport and are essentially a measure of the connectedness of the airport, considering not just the one-hop immediate flight, but also transfers through a multi-hop network (figure 2).

### 3.4. Statistical analysis of correlation

#### 3.4.1. Associating airport connectivity with universities

**Figure 3.**
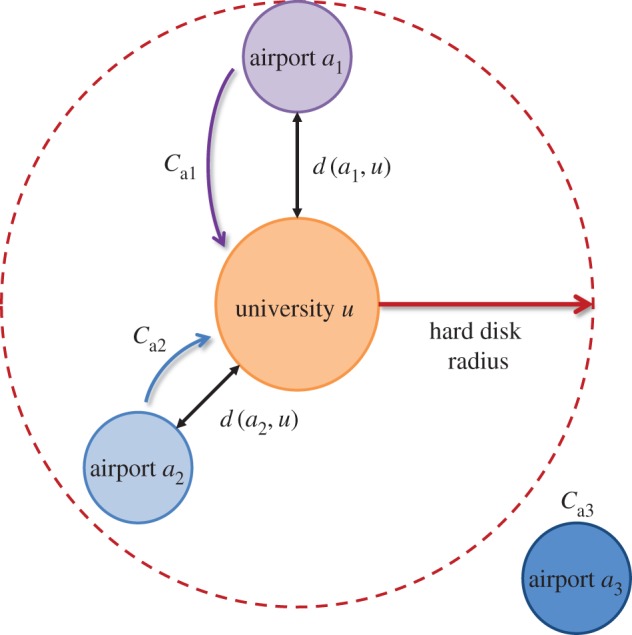
Connectivity association of university to regional airport hubs. Hard disc model which aggregates the distance weighted centrality measure *C*_*a*_ of airports within a finite radius *D*.

Each university is associated with one or more local airports in the vicinity of the city. As a university *i* may have access to a multitude of airports (i.e. from helicopter pads to global airports), we aggregate all the centrality measures within a hard disc radius *D* of the university (see figure 3), such that each university has a connectivity value of

3.3 $$C_{i}(d_{a,i})=\sum_{a_{\in }A}C_{a}d{\left(a,i\right)}^{-\alpha }\;\text{for }d(a,i)<D,$$

where each airport’s centrality contribution is discounted by a distance decay factor *d*(*a*,*i*)^−*α*^, which for entropy maximizing gravity laws is generally *α*=2. As such, we believe that *D* is unnecessary owing to the aggressive distance decay exponent *α* which makes the contribution of distant airports negligible. The aggregated centrality data for each university is given in our dataset—available at [20]. For the purpose of this paper, we will initially investigate the results for a 2 h drive (*D*=100 *km*) and then compare with removing the hard disc distance constraint, which made no significant difference.

#### 3.4.2. Testing against city economic growth as a control variable

A control (confounding) variable is often defined as a variable that correlates, either positively or negatively, with both the dependent and independent variable [25]. In the context of this research, we are aware that given a university’s ranking and its local transport infrastructure connectedness could both be affected by the economic growth of the city to which it is closest. As a result, testing and subsequently controlling for such a confounding variable is necessary in order to obtain valid relational results between the university ranking performance and its global connectivity. Mostly, there are two options for dealing with confounding factors in the analysis stage: *stratification* and *multivariate* methods [26]. However, given the inadequacy of stratified analysis when dealing with confounding factors that have a large number of possible values, we perform an analysis of covariance (ANCOVA). In so doing, by dividing the city economic growth variable into intervals and using those as factor variables, we are able to adjust the comparisons between groups of universities that belong to cities which have experienced a similar economic growth during the years and for which the ranking variable is available. Yet, ANCOVA requires the following assumption to hold (i) the relationship between the dependent variable and the covariate is linear; (ii) regression slopes are homogeneous, i.e. parallel; and (iii) the confounder and independent variable are independent [27]. Thus, if any of those assumptions does not turn out to hold for our data, we propose to use logistic regression to tackle the problem instead.

## 4. Results

### 4.1. Overall ranking trend correlations

As a first step, we have performed two simple overall ranking trend correlations analyses: one for those universities which were consistently ranked top 100 in the ARWU, and another for those universities which were consistently ranked below the 100th in the ARWU from 2005 to 2016. As described in the Methods section, in order to capture the trend, we have computed the *γ* metric as defined in equation (3.2) for each institution. We have then correlated (Pearson’s correlation) the value of the metric across the years with the ranking of the underlying institution. For those universities where the fine grained ranking was available, that is for the top 100, we have correlated the *γ*’s with the overall ranking position (table 2).

**Table 2.** Overall ranking trend correlations: top 100 institutions.

sorted by	degree	*W*_degree_	betweenness	closeness	eigen
overall ranking	−0.04448	−0.04148	−0.15828	0.02114	−0.10894

At the same time, universities ranked from 101 to 500 were sorted by the score on the five variables taken into consideration by ARWU (Alumni, Award, HiCi, NS, PUB and PCP) and reported the correlations associated to the five ways of sorting (table 3).

**Table 3.** Overall ranking trend correlations: bottom 400 institutions.

sorted by	degree	*W*_degree_	betweenness	closeness	eigen
score on alumni	−0.03290	−0.05570	−0.00361	−0.02914	−0.02401
score on award	−0.04409	−0.06154	0.05281	−0.03976	−0.03374
score on HiCi	−0.06901	−0.05078	0.00975	−0.03836	−0.05309
score on NS	−0.16380	−0.02840	−0.01059	−0.02390	−0.15584
score on PUB	−0.03620	−0.01476	−0.00474	−0.02859	−0.01814
score on PCP	−0.06347	−0.04681	0.02995	−0.02332	−0.03339

**Figure 4.**
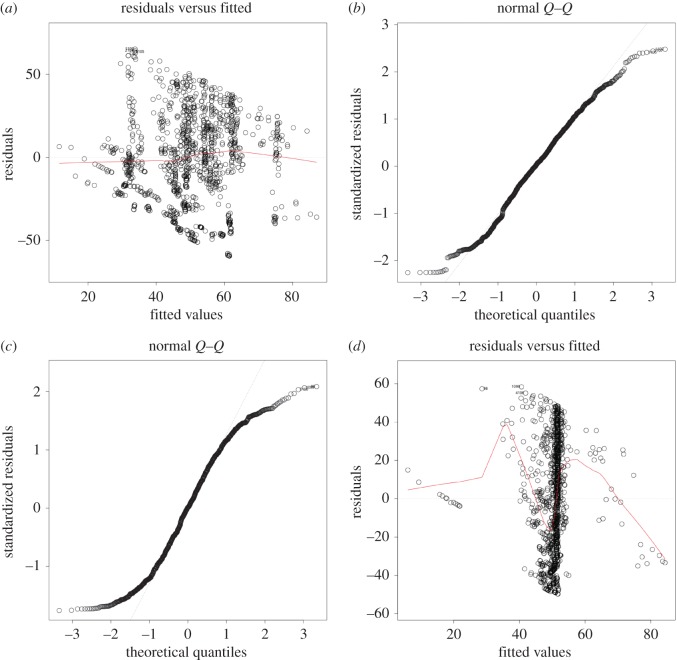
(*a*,*b*) The residuals and *Q*–*Q* plots for rank∼degree+*W*_degree_+ betweenness+closeness+eigen+GaWC_*score*. (*c*,*d*) The residuals and *Q*–*Q* plots for rank ∼ degree + *W*_degree_ + betweenness + closeness + eigen.

Both tables 2 and 3 suggest that this simple analysis does not show any correlation between a university world rank position as given by the ARWU ranking and the metrics associated with the air-transportation network. The same is true for the ANCOVA analysis presented below. Residuals are plotted in figure 4.

Table 4 also shows no correlation between the top 100 universities’ rankings and transport network obtained using ANCOVA analysis.

**Table 4.** ANCOVA results: top 100 institutions.

model 1	rank ∼ degree+*W*_degree_+betweenness+closeness+eigen+GaWC_score					
model 2	rank ∼ degree+*W*_degree_+betweenness+closeness+eigen					
	res.d.f.	d.f.	RSS	sum sq.	*F*-value	*Pr*(>*F*)
1	1186					
2	1197	960290	−11	−131051	17.039	<2.2×10^−16^

### 4.2. Absolute ranking correlations

Yet, results presented in tables 2–4 may be owing to the fact that (i) we are using a rather simplistic correlation analysis and (ii) we are splitting the dataset into two parts: top 100 universities versus bottom 400 universities.

In order to be able to address potential drawback of our initial overall trend analysis, we conduct a more sophisticated interval regression analysis where: (i) the absolute university rank ($M_{\tilde{i},k}$ in equation (3.1)) is the dependent variable (taken in the form of scalar for the top 100 universities and as an interval for the bottom 400); (ii) airport network parameters are the independent variables; (iii) the economic performance of the nearest city is a control (confounding) variable; and (iv) standard errors are clustered at the university level (our analysis assumes that standard errors for each university are correlated over the years). Results of the clustered interval regression are presented in table 5.

**Table 5.** Results of clustered interval regression: university ranking is a dependent variable. (*Significant at 5% level; **significant at 1% level; ***significant at 0.1% level.)

variables	model 1 coeff.	model 1 robust s.e.	model 2 coeff.	model 2 robust s.e.
degree	6.3853	4.5259	5.3839	4.5460
*W*_degree_	−0.0002	0.0002	−0.0001	0.0002
betweenness	1018.2510**	416.9431	948.3942*	422.0244
closeness	−0.0032*	0.0016	−0.0027	0.0016
eigen	−2683.9190	1469.0580	−2357.0660	1476.3020
const.	242.7824***	6.3126	254.6299***	9.9100
GaWC	—	—	−2.4466	1.6202
sigma	139.8764	2.5185	139.6062	2.5130

We find that each university *i*’s ranking in each year *k* has (i) a positive correlation with the aggregate local airports’ betweenness $C_{i,b}$ (measure of hub—equation (3.3)) with a significance of 1–5% and (ii) no correlation with aggregate local airports’ weighted degree $C_{i,w}$. In checking confounding variables, we found that local city economic performance *c*_*i*_ is not a statistically significant influencing factor. Overall, table 5 suggests that the *betweenness* variable is a very important determinant of university ranking. Specifically, a unit increase in *betweenness* leads to at least 948 increase in a university’s world rank. Interestingly, in model 1 (without controlling for GaWC score), the *closeness* variable is also significant at 5% level. However, this effect is very small (a unit increase in *closeness* leads to 0.003 decrease in the university world ranking) and it disappears in model 2 when we control for the economic growth.

**Figure 5.**
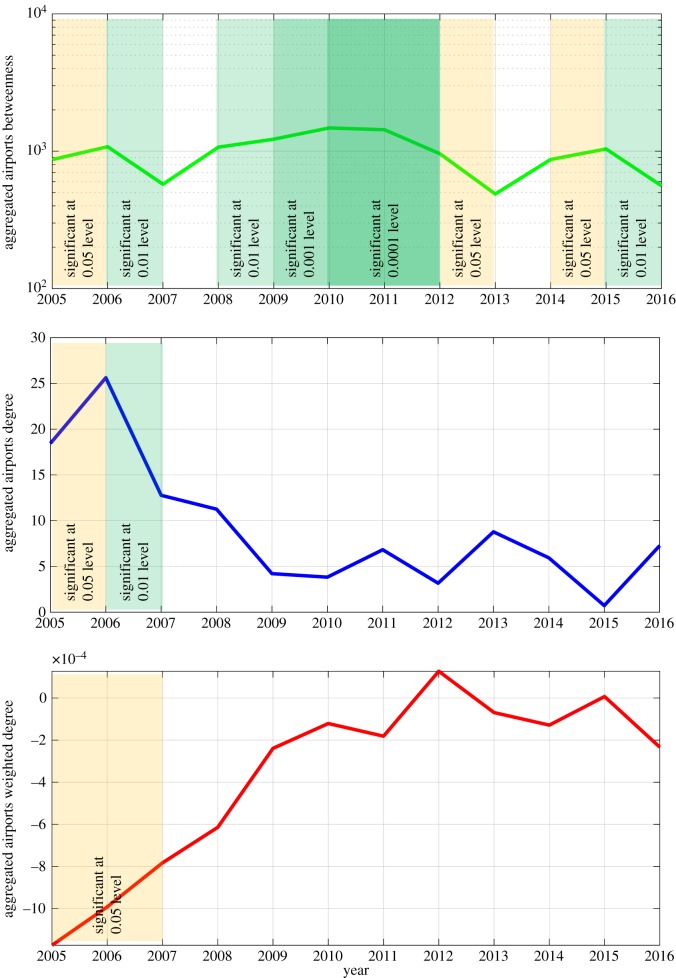
Determinants of university rankings from 2005 to 2016.

We also conduct a series of regressions which aim to predict university world ranking in each year of our analysis (from 2005 to 2016). Results of this analysis are presented in figure 5, which shows three variables: *betweenness*; *degree*; and *weighted degree* which have significant effect on university rankings for at least 2 of 12 years considered in our analysis. In figure 5, years are shown on the horizontal axis and the values of coefficients are shown on the vertical axis. Significance at at least 5% level and at most 0.001% level is indicated by the dashed red lines. Figure 5 shows that *betweenness* is the most robust determinant of university rankings: coefficients are very stable and significant for the majority of years between 2005 and 2016. Two other variables—*degree* and *weighted degree* show a significant impact on university ranking in 2005 and 2006 but starting from 2007 their influence fades away. In 2005 and 2006, the effect of *degree* is positive and large (one unit change in *degree* leads to more than 10 change in world ranking; while the effect of *weighted degree* is very small and negative).

## 5. Discussion

The performance of many aspects of a university is closely related to its ability to interact at an international level. Yet, the strength of international air transport connectivity and the academic performance of the university has not, to our knowledge, been explored until now. In this paper, we set out to quantify the effect of air transport connectivity on local university ranking performance.

We used both the general trend of rankings (§4a) as well as their absolute rankings (§4b) to analyse the correlation. There was a discrepancy in the results, whereby the former yielded no clear correlation and the latter method yielded a clear correlation. One possible explanation of the discrepancy in the results of the two models resides in how they were trained: while the former overall ranking trend correlation model had to be applied to the top 100 and bottom 400 institutions separately, the latter interval regression model was trained on the whole ranking system. Further, when the overall ranking trend correlation model was trained on the bottom 400 institutions, we had to introduce synthetic fine-grained rankings based on the scores the institutions had received in the indicators, which introduced further noise into the data.

The interval regression analysis showed that certain aspects of air transport connectivity are closely correlated with ranking changes. While correlation cannot mean causality, we encourage researchers to use methods such as directed entropy to test the strength of causal arguments. This would involve sampling the probability space of airport and university rankings. Nonetheless, using the results presented in this paper, we can show that the most likely confounding variable (economic output of the city) was not significant in determining the fluctuations in airport hub factor nor the local university rankings. As such, we have a small degree of confidence in saying that, while there may be hidden factors related to the culture and fame of a location, they would require more detailed qualitative analysis.

In terms of impact, if our analysis holds any truth, then the research can inform policy at both the university and the local authority level. At the university level, it is possible to incentivise academics to connect nationally and internationally more. At the regional level, there is growing recognition that universities not only serve as strong economic sources, but are also as part of the intellectual culture and education system. As such, the evidence presented in this paper can inform regional investment policies on improving global connectivity by reducing air transport tax and improving its infrastructure.

## Footnotes

1 According to the Guinness World Records Book, University of Karueein, founded in AD 859 in Fez, Morocco, is the oldest still operating educational institution in the world. In Europe, the oldest university is the University of Bologna, which was founded in 1088.

2 See http://www.lboro.ac.uk/gawc/ for more details on the GaWC city ranking.

3 In stage 1 of the registered report, we originally stated that we use data from 2003 to 2016. However, after data mining, we found that ARWU omitted or merged some of the ranking meta-scores for universities in 2003–2004. This was needed in order to differentiate universities with the same clustered ranking. As such, we have limited our analysis to data from 2005 to 2016 in the stage 2 of this article.

4 See https://developers.google.com/maps/documentation/geocoding/intro.

5 Network-level data are available from the authors upon request. While the network-level data is open, the detailed commercial data (i.e. seat classifications and flight frequency variations per week), which is not used for this paper, will not be made available.

6 Initially, we tried to consider all the permutations of the clustered entries, however, we quickly discovered that given the size of the dataset, this was not technically feasible.

## Data accessibility

The University and Global Cities rankings databases are freely available online for non-commercial usage. The air transport data are purchased from commercial vendor OAG and its aggregated network level data and airport location data along with university ranking data are made available on Dryad: http://dx.doi.org/10.5061/dryad.fv5mn [20].
